# Fluorogenic Assay for Inhibitors of HIV-1 Protease with Sub-picomolar Affinity

**DOI:** 10.1038/srep11286

**Published:** 2015-08-11

**Authors:** Ian W. Windsor, Ronald T. Raines

**Affiliations:** 1 Department of Biochemistry University of Wisconsin–Madison, Madison, WI, 53706, USA; 2 Department of Chemistry, University of Wisconsin–Madison, Madison, WI, 53706, USA

## Abstract

A fluorogenic substrate for HIV-1 protease was designed and used as the basis for a hypersensitive assay. The substrate exhibits a *k*_cat_ of 7.4 s^−1^, *K*_M_ of 15 μM, and an increase in fluorescence intensity of 104-fold upon cleavage, thus providing sensitivity that is unmatched in a continuous assay of HIV-1 protease. These properties enabled the enzyme concentration in an activity assay to be reduced to 25 pM, which is close to the *K*_d_ value of the protease dimer. By fitting inhibition data to Morrison’s equation, *K*_i_ values of amprenavir, darunavir, and tipranavir were determined to be 135, 10, and 82 pM, respectively. This assay, which is capable of measuring *K*_i_ values as low as 0.25 pM, is well-suited for characterizing the next generation of HIV-1 protease inhibitors.

HIV-1 protease is a dimeric, aspartic acid protease. This enzyme is not only an important target for chemotherapeutic agents but also has been a key model for the development of structure-based drug design and in studies of drug resistance^1^,^2^,^3^. Hundreds of small molecules that bind to the enzymic active site and inhibit proteolyic activity have been characterized, ten of which have achieved regulatory approval and clinical relevance for the treatment of HIV/AIDS. Three inhibitors in particular, amprenavir, darunavir, and tipranavir, have garnered significant attention due to their picomolar inhibition constants and reduced susceptibility to drug-resistance mutations in the viral genome^4^,^5^,^6^,^7^,^8^,^9^.

Different workers have used different assays to assess inhibition by tight-binding inhibitors of HIV-1 protease. The ensuing values of inhibition constant (*K*_i_) for the same inhibitor range over several orders-of-magnitude (Table 1). These discrepancies, along with the pending emergence of even more potent inhibitors^10^ requiring even more sensitive characterization techniques, provided an impetus for our work.

Solution-phase assays based on enzymatic activity are the oldest and still the most popular approach for characterizing the inhibition of HIV-1 protease^11^,^12^. The loss of enzymatic activity upon addition of inhibitor is employed as a metric to assess the formation of an enzyme·inhibitor complex. Classical methods employ graphical analysis to estimate a value of IC_50_ and its conversion to a value for the inhibition constant, *K*_i_^13^. This approach is limited to assay conditions in which the *K*_i_ value is above or near the enzyme concentration.

High-affinity inhibitors possess *K*_i_ values below the usable enzyme concentrations of traditional assays. As little free inhibitor is present under sub-saturating conditions, the observed enzymatic activity decreases linearly with inhibitor concentration. This high-affinity inhibitor problem was remedied by curve fitting as described by Morrison^14^, but a computational analysis has revealed that the reliability of his methodology is limited to the determination of *K*_i_ values that are no less than 100-fold below the enzyme concentration^15^.

An alternative assay for characterizing high-affinity inhibitors of HIV-1 protease is isothermal titration calorimetry (ITC)^16^,^17^. ITC is made powerful by its label-free nature and provision of a full thermodynamic characterization. Still, ITC has notable disadvantages compared with activity-based assays. For example, ITC has a theoretical limit in the low nanomolar range for the direct measure of an equilibrium disassociation constant (*K*_d_).

The key to assessing the next generation of HIV-1 protease inhibitors is reducing the enzyme requirement in activity-based inhibition assays. Towards that end, we report here on the design and characterization of a novel fluorogenic substrate for HIV-1 protease. Its attributes—high *k*_cat_ and *k*_cat_/*K*_M_ values and high signal-to-noise ratio—are unprecedented, and enable the rapid, facile determination of sub-picomolar values of *K*_i_.

## Results

### Substrate Design

The first major design criterion for an improved fluorogenic substrate was identifying a peptide that was bound by HIV protease with high affinity and cleaved rapidly in a catalytic manner. To meet this criterion, we employed a peptide substrate that had been selected by phage display. The sequence GSGIFLETSL was reported to have *k*_cat_/*K*_M_ = 1.3 μM^−1^s^−1^ for cleavage between the phenylalanine and leucine residues^18^. These values were determined by a discontinuous HPLC method. Under the same conditions, an endogenous cleavage site in the HIV polyprotein has *k*_cat_/*K*_M_ = 0.022 μM^−1^s^−1^ 18.

**Figure 1:**
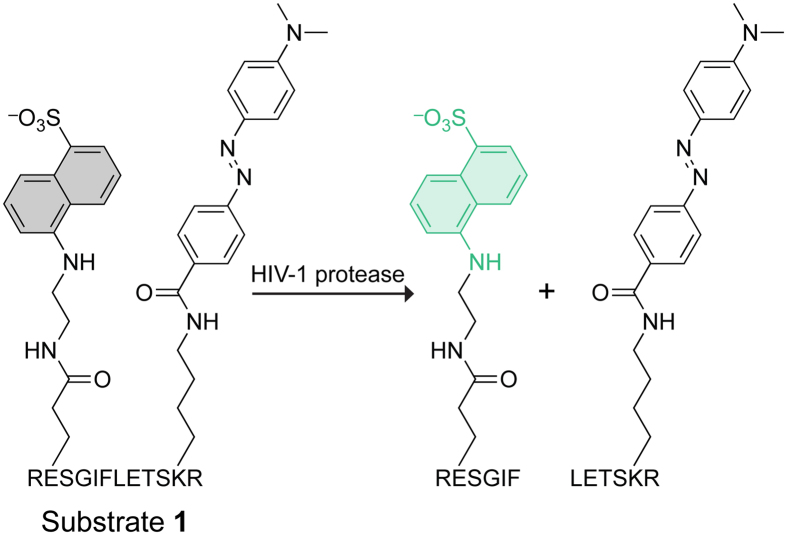
Structure of Substrate 1. Substrate **1** includes EDANS and DABCYL on opposite sides of the scissile bond. Hydrolysis catalyzed by HIV-1 protease relieves quenching by the DABCYL moiety, enabling quantitation of the product (which contains the EDANS moiety) with fluorescence spectroscopy.

The second major design criterion was employing a sensitive method to detect substrate turnover in a continuous manner. We focused on the loss of Förster resonance energy transfer (FRET), which underlies many useful assays^19^, and considered three donor/acceptor moieties. Appending the fluorophore *p*-aminonitrobenzoic acid (Abz) and installing a nitro group in the *para* position of phenylalanine as a quenching chromophore is the basis for a popular HIV protease substrate^11^. This pair, however, lacks sensitivity, as Abz is a weak fluorophore and its use provides a relatively low signal-to-noise ratio. FRET between fluorescein and rhodamine is the basis for some of the most sensitive known assays for enzymatic activity^20^, but protonation of a fluorescein moiety at pH 5, which is optimal for catalysis by HIV-1 protease^12^, compromises the utility of this pair for our purpose, and others^21^,^22^. We chose 5-((2-aminoethyl)amino)naphthalene-1-sulfonic acid (EDANS) and 4-(4-dimethylaminophenylazo)benzoic acid (DABCYL) as a FRET pair^12^,^20^. We installed these moieties into GSIFLETSL by replacing the glycine residue at the N-terminus with a glutamate–EDANS conjugate and by replacing the leucine residue at the C-terminus with a lysine–sDABCYL conjugate^12^. Finally, we added an arginine residue to each terminus to enhance aqueous solubility at pH 5, thereby generating substrate **1** (Fig. 1).

### Assay Design

Our assay was designed to minimize the enzyme concentration while maintaining a high signal-to-noise ratio. An inherent complexity is that HIV-1 protease is an obligate dimer. Significant attention has been paid to the *K*_d_ value of the HIV-1 protease dimer, and conflicting ideas abound regarding an appropriate enzyme concentration for activity assays^23^. Freire and coworkers employed a rigorous thermodynamic approach to determine a dimer *K*_d_ value of 23 pM^24^. We used this value as a lower limit for the enzyme concentration in our assays. Additionally, initial velocities require measurements from <10% substrate turnover, and a convenient upper limit for the enzyme concentration in our assays was determined to be 6.5 nM. Upon cleavage by HIV-1 protease, the fluorescence intensity (*I*) of substrate **1** increases by *I*_f_/*I*_o_ = 104.

### Michaelis–Menten Kinetics

**Figure 2:**
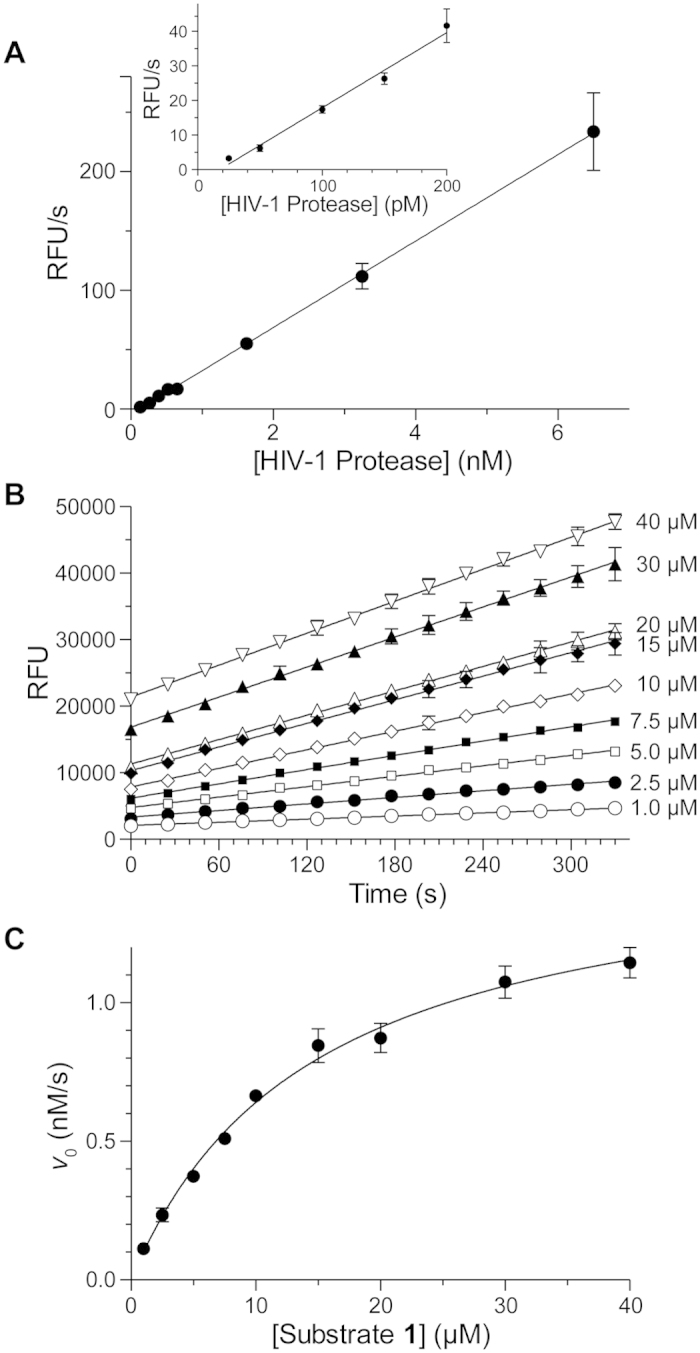
Catalysis of the Hydrolysis of Substrate 1 by HIV-1 Protease. Initial velocities were measured in 50 mM sodium acetate buffer, pH 5.0, containing NaCl (0.10 M), DMF (2% v/v), TFA (0.002% v/v), substrate **1**, and HIV-1 protease. (**A**) Plot of initial velocities at 10 μM substrate **1** and 130 pM–6.5 nM HIV-1 protease (3 replicates with a gain setting of 180). Inset: Plot of initial velocities at 10 μM substrate **1** and 25 pM–250 pM HIV-1 protease (4 replicates). Data were fitted by linear regression. (**B**) Progress curves at 1–40 μM substrate **1** and 214 pM HIV-1 protease (4 replicates). Data were fitted by linear regression to give initial velocities. (**C**) Plot of initial velocities from panel B. Data were fitted by non-linear regression to the Michaelis–Menten equation (eq. 1) to derive the kinetic parameters listed in Table 2.

Michaelis–Menten kinetics were used to evaluate the performance of substrate **1** as a substrate for HIV-1 protease. The initial velocity was directly proportional to enzyme concentration at a fixed substrate concentration (Fig. 2A), and increased with substrate concentration at a fixed enzyme concentration (Fig. 2B). The latter data fitted well to the Michaelis–Menten equation (Fig. 2C). The observed *V*_max_ value of 1.58 nM·s^−1^ for substrate **1** at an enzyme concentration of 214 pM afforded a *k*_cat_ value of (7.4 ± 0.2) s^−1^; the *K*_M_ value was (14.7 ± 1.0) μM (Table 2).

### Determination of *K*_i_ Values with Morrison’s Equation

**Figure 3:**
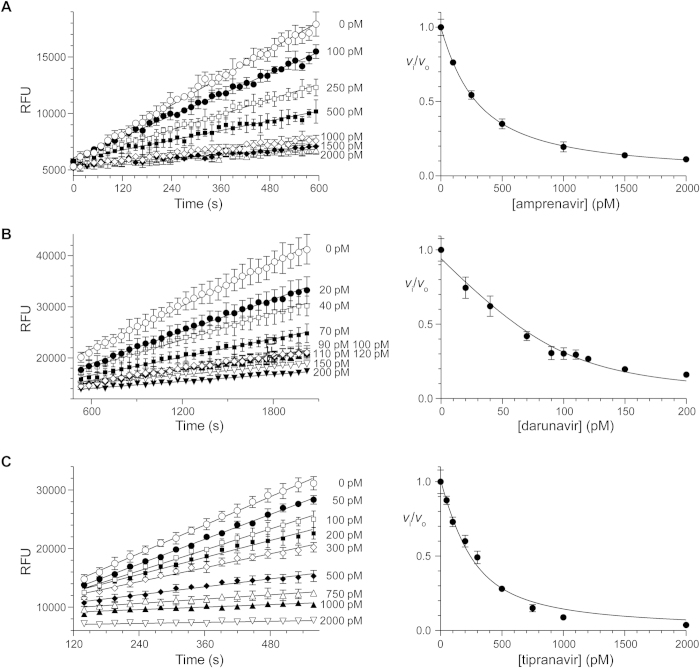
Inhibition of HIV-1 protease by Amprenavir, Darunavir and Tipranavir. Plots showing the inhibition of HIV-1 protease in 50 mM sodium acetate buffer, pH 5.0, containing NaCl (0.10 M), DMF (2% v/v), TFA (0.002% v/v), and substrate **1** (10 μM) by (**A**) amprenavir at 120 pM enzyme (*R*^2^ = 0.99), (**B**) darunavir at 100 pM enzyme (*R*^2^ = 0.96), and (**C**) tipranavir at 200 pM enzyme (*R*^2^ = 0.98) (4 replicates). Data were fitted by non-linear regression to Morrison’s equation (eq. 2) to derive the values of *K*_i_ listed in Table 1.

Substrate **1** was used as the basis for assays of the inhibition of HIV-1 protease by amprenavir, darunavir, and tipranavir (Fig. 3). The data fitted well to Morrison’s equation, and afforded values of *K*_i_ values (Table 1). Darunavir and tipranavir exhibited time-dependent inhibition; amprenavir did not. Pre-equilibrium data were omitted from the initial-velocity data fitted by linear regression.

## Discussion

An assay of high sensitivity is critical for assessing the efficacy of high-affinity inhibitors of enzymatic activity. The HIV-1 protease substrate described herein provides initial velocity data of unmatched quality and sensitivity. Substrate **1** has a 1.5-fold higher *k*_cat_ value, 7-fold lower *K*_M_ value, and higher signal-to-noise ratio than does the parent substrate developed by Matayoshi and coworkers (Table 2)^12^. The Abz-based substrate developed by Toth and Marshall has a similar *K*_M_ value, though substrate **1** provides a 17-fold greater signal-to-noise ratio^11^. These improvements in kinetic and spectroscopic parameters have enabled us to reduce the concentration of enzyme in standard assays to values close to the *K*_d_ value of dimeric HIV-1 protease.

To quantify the utility of a substrate, we define the sensitivity (*S*) of an assay as the increase in fluoresence intensity brought about by the action of an enzyme on a low concentration of substrate. We express sensitivity (*S*) as the product of the kinetic parameter *k*_cat_/*K*_M_ and the spectroscopic parameter *I*_f_/*I*_o_: *S* = (*k*_cat_/*K*_M_)(*I*_f_/*I*_o_). By this measure, the sensitivity of an assay that uses substrate **1** is ≥10-fold greater than any fluorescence-based assay for HIV-1 protease activity (Table 2).

The values of *K*_i_ for amprenavir, darunavir, and tipranavir derived from initial velocities for the cleavage of substrate **1** are consistent with literature values (Table 1). Notably, the *K*_i_ value for darunavir determined herein is comparable to literature values and much closer to values reported by other activity-based methods than is the value determined by an indirect, competitive displacement method^10^, which is two orders of magnitude lower. The high signal-to-noise, low variation in initial velocity measurements and low standard error for fits of inhibition data by Morrison’s equation makes substrate **1** a useful probe for assaying high-affinity inhibitors.

According to a computational assessment, Morrison’s equation can be used to determine values of *K*_i_ that are up to 100-fold lower than the concentration of enzyme in an assay^15^. Because enzyme concentrations as low as 25 pM provided valuable data herein, we believe that our assay can be used to determine *K*_i_ values that are ≥250 fM. We anticipate the use of substrate **1** in the development of the next generation of HIV-1 protease inhibitors.

## Methods

### Materials

The HIV-1 protease inhibitors darunavir (from Tibotec, Inc), amprenavir, and tipranavir were obtained through the AIDS Reagent Program, Division of AIDS, NIAID, NIH. Substrate **1** was synthesized and purified by HPLC to 99.5% by Biomatik (Wilmington, DE). All inhibitors and peptides were used without further purification.

### Plasmid Preparation

Double-stranded DNA encoding a pseudo–wild-type HIV-1 protease and flanked by regions of homology near the T7 promoter and terminator found in the pET32b vector was obtained from IDT (Coralville, IA). This HIV-1 protease had Q7K, L33I, L63I, C67A, and C95A substitutions^25^. Linear pET32b was prepared by PCR using primers that were the reverse complements of the DNA encoding HIV-1 protease. Gene and plasmid fragments were combined with Gibson assembly^26^.

### Protein Purification

BL-21 codon-plus RIL from Agilent Technologies (Santa Clara, CA) was transformed freshly with the pET32b-HIV protease. A single colony was used to inoculate 1 L of Luria–Bertani medium containing ampicillin (200 μM) in a Fernbach flask shaken at 37 °C. Expression was induced by the addition of IPTG (to 2 mM) upon reaching saturation (*OD*_600 nm_ 2.8–3.4), and the culture was grown for an additional hour. HIV-1 protease was purified and folded as described previously^27^. Cells were pelleted, resuspended in 20 mM Tris–HCl buffer, pH 7.4, containing EDTA (1 mM) and lysed at 18 kPSI using a cell disruptor from Constant Systems (Kennesaw, GA). Inclusion bodies were isolated by centrifugation at 10,000 *g* for 10 min. The pelleted inclusion bodies were washed with resuspension buffer containing urea (1.0 M) and Triton X-100 (1% v/v), and again with resuspension buffer. Inclusion bodies were isolated by centrifugation and lyophilized.

Inclusion bodies were dissolved by sonication in aqueous acetic acid (50% v/v) at a concentration of 5 mg/mL. The solution was clarified by centrifugation, and soluble protein was applied to a Superdex 75 gel-filtration column from GE Healthcare Bio-Sciences (Pittsburgh, PA) that had been pre-equilibrated with aqueous acetic acid (50% v/v). Unfolded HIV-1 protease that eluted as major peak near one column-volume was pooled and lyophilized. HIV-1 protease was folded at a concentration of 0.1 mg/mL in 100 mM sodium acetate buffer, pH 5.5, containing ethylene glycol (5% v/v) and glycerol (10% v/v). The solution of folded HIV-1 protease was clarified by centrifugation and concentrated with an Amicon stirred-cell concentrator equipped with a 10 K MWCO membrane from EMD Millipore (Billerica, MA). The concentrated protease was applied again to a Superdex 75 gel-filtration column that had been pre-equilibrated with the folding buffer. A new major peak containing dimeric HIV-1 protease was pooled and concentrated. The folding buffer was exchanged for 1 mM sodium acetate buffer, pH 5.0, containing NaCl (2 mM) using a PD-10 desalting column. A solution (~1.5 mg/mL) of purified HIV-1 protease was flash-frozen in liquid nitrogen and stored at −80 °C until use.

### Enzymatic Activity Assays

Substrate **1** was dissolved at a concentration of 1.0 mM in DMF containing TFA (0.1% v/v). Fluorescence of the EDANS moiety was measured on a M1000 Pro plate reader from Tecan (Maennedorf, Switzerland) by excitation at 340 nm and observation of emission at 490 nm. A fluorophore calibration was performed to enable quantitation of assay data. The product exhibits a fluorescence of 70 RFU/nM at a gain setting of 216, and all assays were performed at this gain setting unless indicated otherwise. Assays were performed in a Corning black, flat bottom, non-binding surface, 96-well plate. Assays were conducted at room temperature in 200 μL of 50 mM sodium acetate buffer, pH 5.0, containing NaCl (0.10 M), DMF (2% v/v), substrate **1** (1–40 μM), and HIV-1 protease (25 pM–6.5 nM). Assays with 30 and 40 μM of substrate **1** required 3% and 4% v/v DMF, respectively. Inhibition assays were conducted with picomolar–nanomolar inhibitor (depending on the enzyme concentration and *K*_i_ value) and 10 μM substrate **1**. Inhibition assays were monitored for until ≤7% of the substrate was converted to product. Initial velocities were measured in quadruplicate.

Solution concentrations of HIV-1 protease (10.7 kDa) was determined by measuring the absorbance at 280 nm and estimating the extinction coefficient as 12,500 M^−1^cm^−1^ with software from ExPASy^28^. The fraction of active enzyme was determined by active-site titration using darunavir as the titrant and found to be 76% with respect to the value based on the *A*_280 nm_. Fluorescence was monitored over the linear range of the detector, which corresponds to 700 nM of product formation at a gain setting of 216.

### Data Analysis

The velocity (*v*) of all enzyme-catalyzed reactions was obtained by linear fit of initial-velocity data using Prism 6 software from Graphpad (La Jolla, CA). Pre-equilibrium values from the beginning of data sets were removed to provide fluorescence measurements that were linear as a function of time (Fig. 3).

Values of *v* in the absence of an inhibitor were fitted to the Michaelis–Menten equation (eq. 1) by non-linear regression using Prism 6 software.


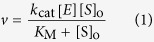


In eq 1, [S]_o_ refers to the concentration of substrate **1** prior to the addition of enzyme.

Values of *v* in the presence of an inhibitor were fitted to Morrison’s equation (eq. 2) by non-linear regression using Prism 6 software.


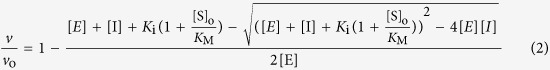


In eq 2, *v*_o_ refers to the reaction in the absence of inhibitor. Enzymatic activity measured in the absence of an inhibitor was used to determine the enzyme concentration for data obtained in the presence of an inhibitor. These enzyme concentrations, which agreed (±10%) with values estimated by active-site titration, were used as constraints for the non-linear regression analysis.

## Additional Information

**How to cite this article**: Windsor, I. W. & Raines, R. T. Fluorogenic Assay for Inhibitors of HIV-1 Protease with Sub-picomolar Affinity. *Sci. Rep.* **5**, 11286; doi: 10.1038/srep11286 (2015).

## Figures and Tables

**Table 1:** Inhibition constants (pM) reported for amprenavir, darunavir, and tipranavir.

Assay type	Amprenavir	Darunavir	Tipranavir	Reference
Enzymatic activity	100	8	88	8
(Unknown)	600	—	—	4
Enzymatic activity	—	—	8	5
(Unknown)	—	16	—	7
ITC (competitive displacement)	390	4.5	—	6
fluorescence (competitive displacement)	36	0.147	—	10
Enzymatic activity	57	—	—	16
Enzymatic activity	135 ± 6	10 ± 1	82 ± 6	This work

**Table 2:** Kinetic parameters of popular HIV-1 protease substrates and substrate 1.

Substrate	*k*_cat_ (s^−1^)	*K*_M_ (μM)	*k*_cat_/*K*_M_ (μM^−1^s^−1^)	*I*_f_/*I*_o_	*S*^a^ (μM^−1^s^−1^)
DABCYL-SQNYPIVQ-EDANS^b^	4.9 ± 0.2	103 ± 8	0.048 ± 0.004	40	2
Abz-TINleF(*p*-NO_2_)QR	8.2 ± 0.4^c^	13 ± 1^*c*^	0.63 ± 0.06	6^d^	4
Substrate **1**^e^	7.4 ± 0.2	14.7 ± 1.0	0.50 ± 0.04	104	52

^a^Sensitivity: *S* = (*k*_cat_/*K*_M_)(*I*_f_/*I*_o_).

^b^Values ( ± SD) are from ref. 12.

^c^Values ( ± SE) are from ref. 16.

^d^Value is from ref. 11.

^e^Values ( ± SE) are from this work.
